# *SEC61G* identified as a prognostic biomarker of head and neck squamous cell carcinoma

**DOI:** 10.1007/s00405-021-06955-7

**Published:** 2021-06-25

**Authors:** Yanan Shi, Yibo Liang, Wei Wang, Guimin Zhang

**Affiliations:** 1 Department of Otolaryngology Head and Neck Surgery, Tianjin First Center Hospital, No. 24 Fukang Road, Nankai District, Tianjin, 300192 People’s Republic of China; 2 Institute of Otolaryngology of Tianjin, Key Clinical Discipline of Tianjin (Otolaryngology), Otolaryngology Clinical Quality Control Centre, Tianjin, People’s Republic of China

**Keywords:** *SEC61G*, Head and neck squamous cell carcinoma, TCGA, Prognosis

## Abstract

**Purpose:**

It is of obvious interest to identify clinical prognosis-related oncogenes in HNSCC (head and neck squamous cell carcinoma).

**Methods:**

Based on the available datasets within the TCGA (The Cancer Genome Atlas) and the GEO (Gene Expression Omnibus) databases, the potential mechanism of action of the *SEC61G* (*SEC61* translocon subunit gamma) gene in HNSCC tumorigenesis was explored by several bioinformatics approaches.

**Results:**

There was a higher expression level of *SEC61G* in primary HNSCC tumor tissues than in normal tissues. Moreover, highly expressed *SEC61G* was statistically associated with the poor survival prognosis of HNSCC patients. When HPV (human papilloma virus) was considered, we also observed a relatively lower proportion of “arm-level gain” and “high amplification” types of CNA (copy-number alteration) in the HNSCC-HPV (+) group than in the HNSCC-HPV (−) group. Additionally, we identified *SEC61G* CAN-correlated genes, such as CCT6A (chaperonin-containing TCP1 subunit 6A) and HUS1 (HUS1 checkpoint clamp component), and found a correlation between *SEC61G* copy-number segments and prognosis related to overall and progression-free survival intervals of HNSCC patients. Moreover, the molecular regulation mechanisms of the spliceosome, ribosome, proteasome degradation, cell adhesion, and immune infiltration of B and CD8^+^ T cells may contribute to the involvement of *SEC61G* in the pathogenesis of HNSCC.

**Conclusions:**

The *SEC61G* gene was identified for the first time as a prognostic biomarker of HNSCC. The detailed underlying mechanism merits further research.

**Supplementary Information:**

The online version contains supplementary material available at 10.1007/s00405-021-06955-7.

## Introduction

*SEC61G* is the main member of the Sec61 complex, which is a transmembrane channel across which proteins are translocated and integrated into the ER (endoplasmic reticulum) membrane [1, 2]. *SEC61G* is essential for protein translocation into the mammalian endoplasmic reticulum [2]. The expression of *SEC61G* was reportedly associated with IFN-K immunosuppressive therapy for lupus nephritis [3], while the methylation modification of *SEC61G* was related to Balkan endemic nephropathy [4, 5].

Several publications have reported the correlation between *SEC61G* expression and the pathogenesis of certain cancers and the prognosis of patients with these cancers. For example, the binding of lncRNA LINC02418 and miR-4677-3p (microRNA-4677-3p) was associated with the expression of *SEC61G* in NSCLC (non-small cell lung cancer) cells [6]. The *SEC61G*-*EGFR* fusion gene was detected in the MI (mitogen-independent) stem-like cells of pediatric ependymomas [7]. It was also reported that high expression of the *SEC61G* gene was correlated with a poor prognosis of glioblastoma patients in a study that used the TCGA and CGGA (Chinese Glioma Genome Atlas) datasets [8]. The *SEC61G* gene was identified as a hub molecule of parathyroid adenoma development [9].

In the present study, we first collected the relevant available datasets from the TCGA and GEO databases to explore the oncogenic role of *SEC61G* in HNSCC. A series of analyses, including gene expression, survival prognosis, genetic alteration, gene enrichment, and immune infiltration, were carried out.

## Methods

### Expression feature analysis

After logging into the UALCAN website (http://ualcan.path.uab.edu/analysis-prot.html), entering the gene information, and selecting the cancer type, we obtained the differential expression data of the *SEC61G* gene in normal and HNSCC tissues. The transcript per million was used for the expression level of *SEC61G*. Two factors, namely, human papilloma virus (HPV) infection status and sex, were also considered. In addition, we logged into the Oncomine database (https://www.oncomine.org/resource/main.html) and entered the word “*SEC61G*”. Differential expression data of *SEC61G* between the normal and different tumor tissues were obtained from the GEO database with the threshold of *p* value = 0.05 and fold change = 1.5. A pooled analysis across five analyses was also performed.

### Survival prognosis analysis

We applied the “survival map” module of GEPIA2 [10] to obtain the OS (overall survival) and DFS (disease-free survival) rate significance map data of *SEC61G* for all tumor cases from the TCGA database. A cutoff point of 50% was set to split the high-expression and low-expression cohorts. “Significance Level” was set at 0.01, and “*p* value adjustment” of FDR was used. The survival plots with a log-rank test were also yielded via the “survival analysis” module of GEPIA2. We also considered three factors, race, histological grade, and sex, for the survival analyses. In addition, we utilized the interactive operation interface of the PrognoScan database [11] to conduct an RFS (relapse-free survival) rate analysis of HNSCC patients in the GSE2837 dataset.

### Genetic alteration analysis

We then studied the association between *SEC61G* expression and CNA (copy-number alteration) across all TCGA cancers using the “sCNA” module of TIMER2.0 (http://timer.cistrome.org/). After logging into the cBioPortal web (https://www.cbioportal.org/) [12, 13], we chose the “TCGA PanCancer Atlas studies” in the “Quick select” section and entered “SEC61G” to obtain the association between log2 copy-number values and the mRNA expression Z scores of *SEC61G*. Using a LinkedOmics approach (http://www.linkedomics.org/login.php) [14], we identified the potential *SEC61G* CNA-associated genes. Spearman’s rho statistical analysis was conducted. We also utilized UCSC-Xena (https://xena.ucsc.edu/) to investigate the potential correlation between the copy-number segments of *SEC61G* and the overall survival, disease-specific survival, progression-free interval, and disease-free interval status of the patients. Kaplan–Meier plots with log-rank *p* values were generated.

### Gene enrichment analysis

Next, we performed cluster analysis of the *SEC61G*-correlated significant genes through the LinkedOmics approach [14]. Heat maps and association plots covering the significant genes positively/negatively correlated with *SEC61G* expression are presented. We also probed the expression correlation between *SEC61G* and the selected genes through a Spearman correlation test. Additionally, the GO (gene ontology), KEGG (Kyoto encyclopedia of genes and genomes) pathway, WikiPathway, and GSEA (gene set enrichment analysis) profiles were obtained.

### Immune infiltration analysis

Finally, based on the HNSCC dataset of the TCGA, we investigated the potential association between *SEC61G* expression and the infiltration level of different immune cells using the “immune-gene” module of TIMER2.0 with the algorithms TIMER, EPIC, QUANTISEQ, XCELL, MCPCOUNTER, CIBERSORT, and CIBERSORT-ABS [15].

## Results

### Gene expression

**Fig. 1:**
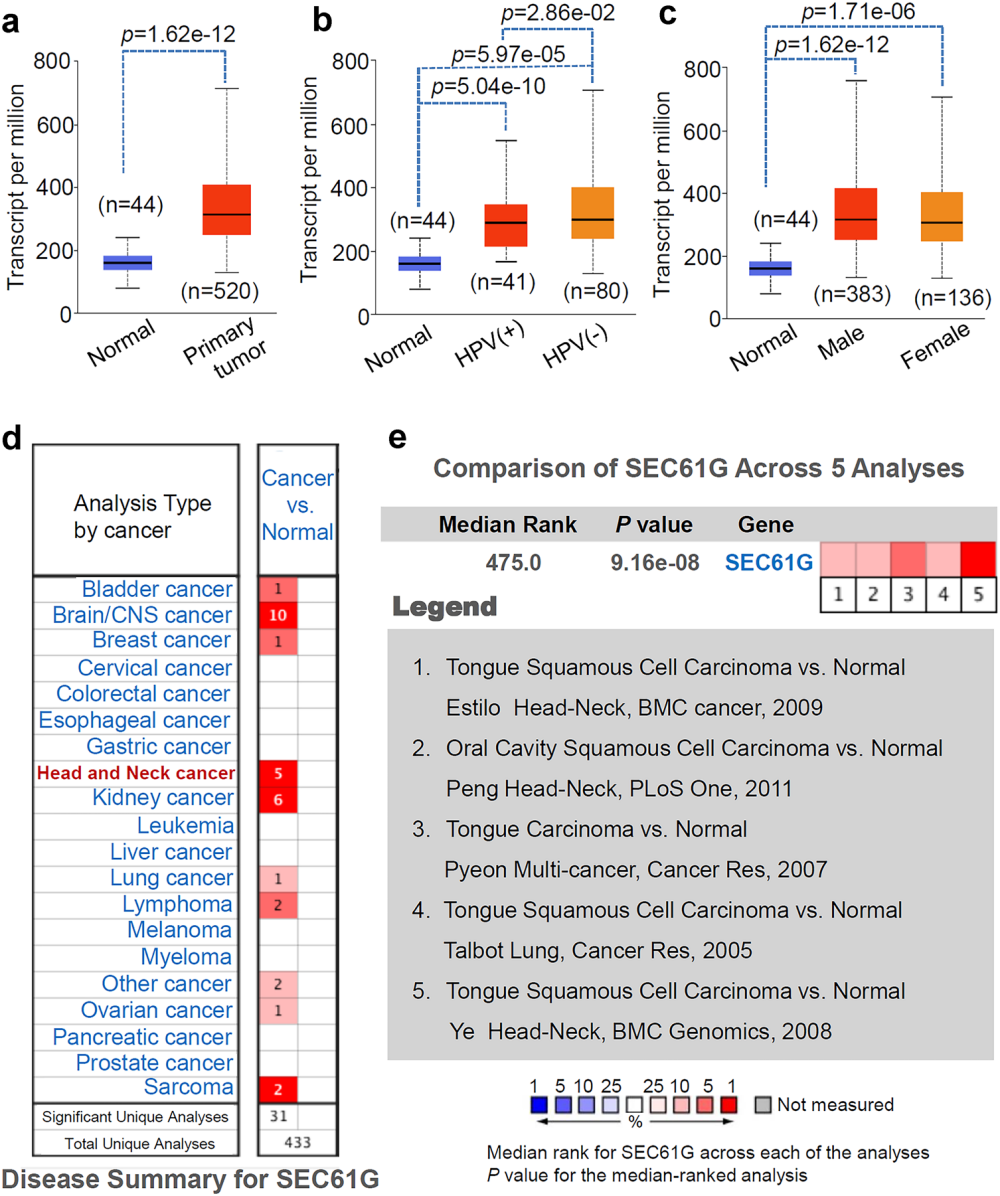
The expression level of the *SEC61G* gene. **a** Expression differences in the *SEC61G* gene in normal and HNSCC tissues were detected based on the HNSCC dataset from the TCGA. Two factors, including, **b** HPV and **c** sex, were considered. **d** Through the Oncomine database, differential expression data of *SEC61G* between normal and different tumor tissues were provided. **e** Pooled analysis of *SEC61G* across five GEO studies was conducted

*SEC61G* structure is conserved across different species (Fig. S1). In the present study, we investigated the possible oncogenic role of human *SEC61G* in HNSCC. We first compared the differential expression of *SEC61G* between normal tissues and HNSCC tissues using two factors (HPV and sex). As shown in Fig. 1a, we observed high *SEC61G* expression in primary HNSCC tumor tissues (*n* = 520) compared with normal tissues (*n* = 44) (*p* = 1.62e-12). Additionally, there was a higher expression level of *SEC61G* in both HNSCC-HPV (+) (Fig. 1b, *p* = 5.04e-10) and HNSCC-HPV (−) (*p* = 5.97e-05) tissues than in controls. The expression of *SEC61G* was higher in the HNSCC-HPV (−) group than in the HNSCC-HPV (−) group (Fig. 1b, *p* = 2.86e-02). Likewise, compared with the normal tissues, we observed a higher expression level of *SEC61G* in both the male (Fig. 1c, *p* = 1.62e-12) and female (*p* = 1.71e-06) groups. However, there was no difference between the male and female groups (Fig. 1c).

Based on the ONCOMINE database analysis, we found that the *SEC61G* gene was highly expressed in many types of tumors, such as brain/CNS cancer, head and neck cancer, and kidney cancer, compared with its expression in normal control tissues (Fig. 1d). We further determined the high expression pattern of *SEC61G* in head and neck cancer using pooled analysis (Fig. 1e, *p* = 9.16e-08).

### Survival analysis

**Fig. 2:**
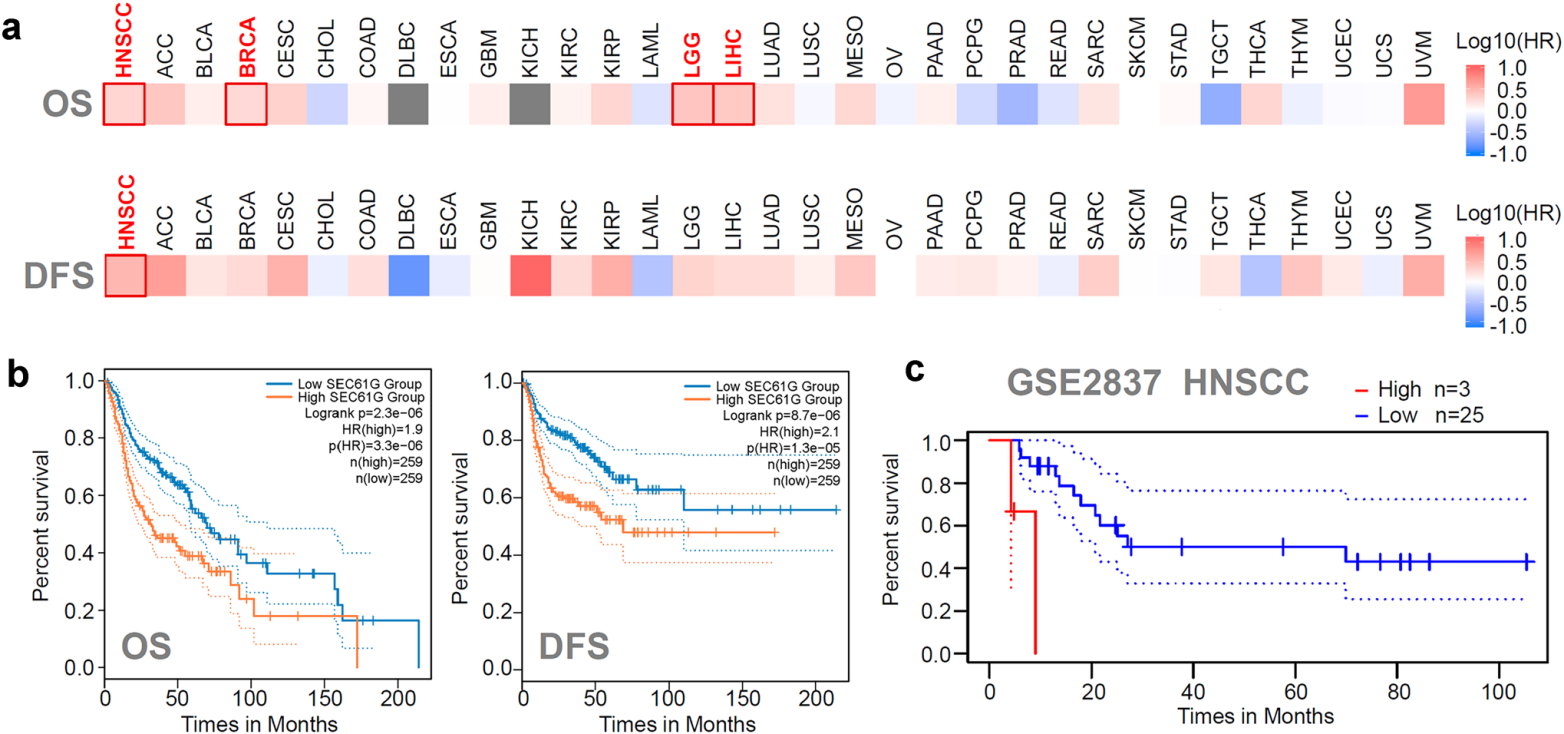
Correlation between *SEC61G* expression and survival prognosis of HNSCC cases. **a** Survival maps of OS and DFS rates were obtained using the GEPIA2 approach. **b** Survival plots of HNSCC were displayed. **c** Based on the GSE2837 dataset, an RFS rate analysis of HNSCC patients was performed

Subsequently, we divided the HNSCC samples into high-expression and low-expression groups according to the expression level of *SEC61G* and investigated the correlation between *SEC61G* expression and the prognosis of patients with different types of cancers, mainly using datasets from the TCGA and GEO databases. As shown in the survival maps in Fig. 2a, we observed that high expression of *SEC61G* was related to a poor OS (overall survival) rate among HNSCC, BRCA (breast invasive carcinoma), LGG (brain lower-grade glioma), and LIHC (liver hepatocellular carcinoma) patients in the TCGA database. DFS (disease-free survival) rate analysis data (Fig. 2a) indicated a correlation between high expression of *SEC61G* and poor prognosis of TCGA HNSCC patients. The detailed OS and DFS rate survival plots for the HNSCC patients are shown in Fig. 2b. We also observed positive correlations (Fig. S2) when considering the other three factors, race (*p* = 0.00079), histological grade (*p* = 0.033), and sex (*p* = 0.0018). Additionally, we detected a poor relapse-free survival rate in HNSCC patients with high SEC61G expression in the GSE2837 dataset (Fig. 2c). These data indicate a correlation between high expression of *SEC61G* and poor survival prognosis in HNSCC patients.

### Genetic alteration analysis

**Fig. 3:**
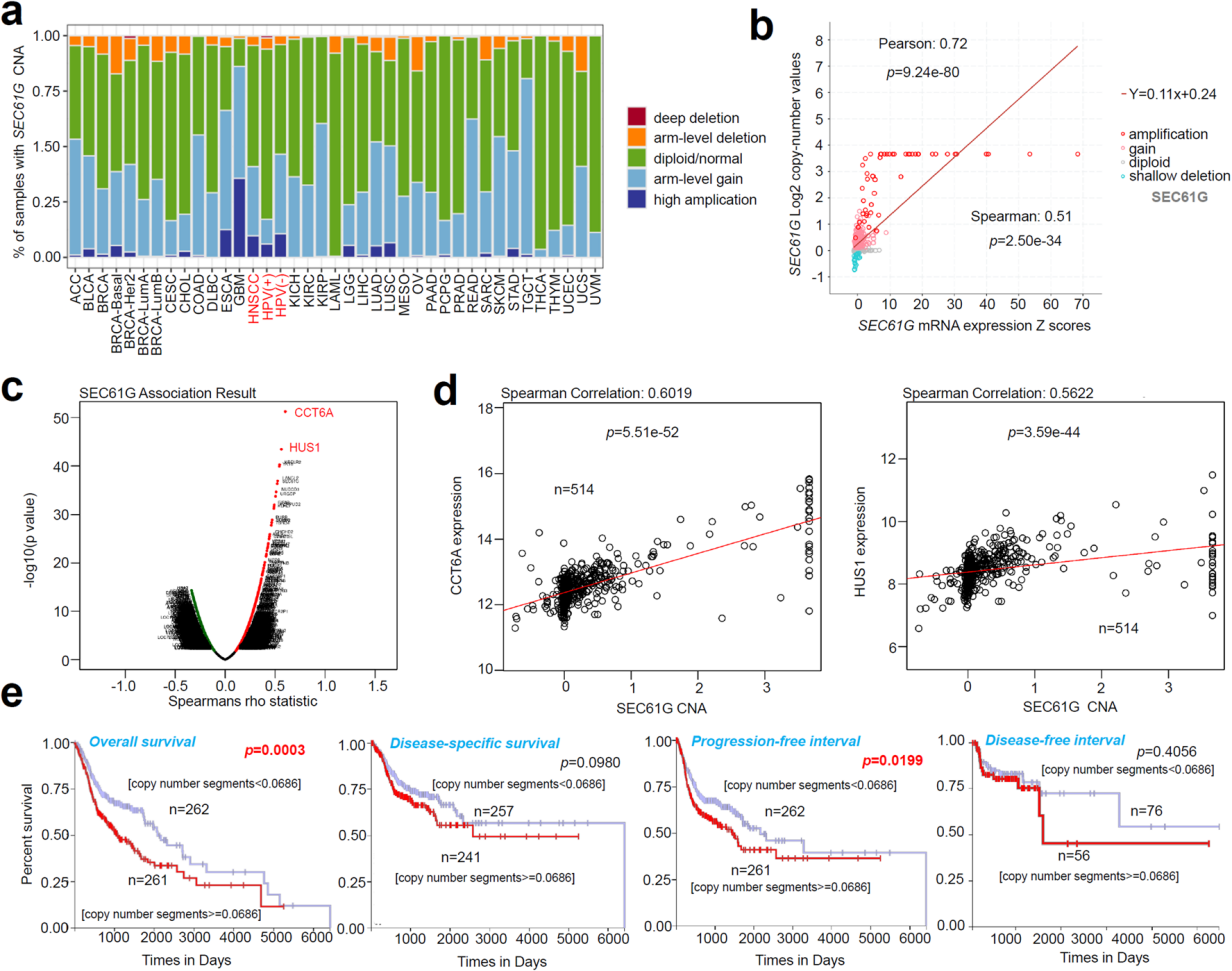
Genetic alteration analysis of *SEC61G*. **a** Association between *SEC61G* expression and CNA across all TCGA tumors was analyzed via TIMER2.0. **b** Correlation analysis between *SEC11G* log2 copy-number values and the mRNA expression Z score was performed via cBioPortal. **c** We identified potential *SEC61G* CNA-associated genes via LinkedOmics. **d** Potential correlations between the copy-number segments of *SEC61G* and the status of overall survival, disease-specific survival, progression-free interval, and disease-free interval rates were determined via UCSC-Xena

The CNA status of *SEC61G* in different tumors in the TCGA database was also analyzed. As shown in Fig. 3a, the “arm-level gain” variant of *SEC61G* showed a higher proportion in TGCT (testicular germ cell tumors), READ (rectum adenocarcinoma), KIRP (kidney renal papillary cell carcinoma), and other tumors. The “high amplification” variant was most common in GBM (glioblastoma multiforme) tumors. In contrast, in the HNSCC, HNSCC-HPV (+), and HNSCC-HPV (−) groups, “arm-level gain” and “high amplification” variants were present at moderate levels. We observed a relatively lower proportion of the two types of CNAs in the HNSCC-HPV (+) group compared with the HNSCC-HPV (−) group. Moreover, we observed a positive correlation between *SEC11G* log2 copy-number values and the mRNA expression Z scores (Fig. 3b, *p* = 9.24e-80, *R* = 0.72 for a Pearson test, *p* = 2.50e-34, *R* = 0.51 for Spearman’s test). Based on the HNSCC dataset of the TCGA, the potential *SEC61G* CNA-correlated genes identified using Spearman’s rho statistical analysis are shown in Fig. 3c. There was a positive correlation between *SEC61G* CNA and the expression level of CCT6A (Fig. 3d, *p* = 5.51e-52, Spearman correlation = 0.6019) and HUS1 (*p* = 3.59e-44, Spearman correlation = 0.5622). In addition, we explored the potential association between the genetic alteration of *SEC61G* and the clinical survival prognosis of TCGA HNSCC patients. We observed a correlation between *SEC61G* copy-number segments and overall survival (Fig. 3e, *p* = 0.0003) and progression-free intervals (*p* = 0.0199) but not with disease-specific survival rates or disease-free intervals in HNSCC patients.

### Enrichment analysis of *SEC61G*-related partners

**Fig. 4:**
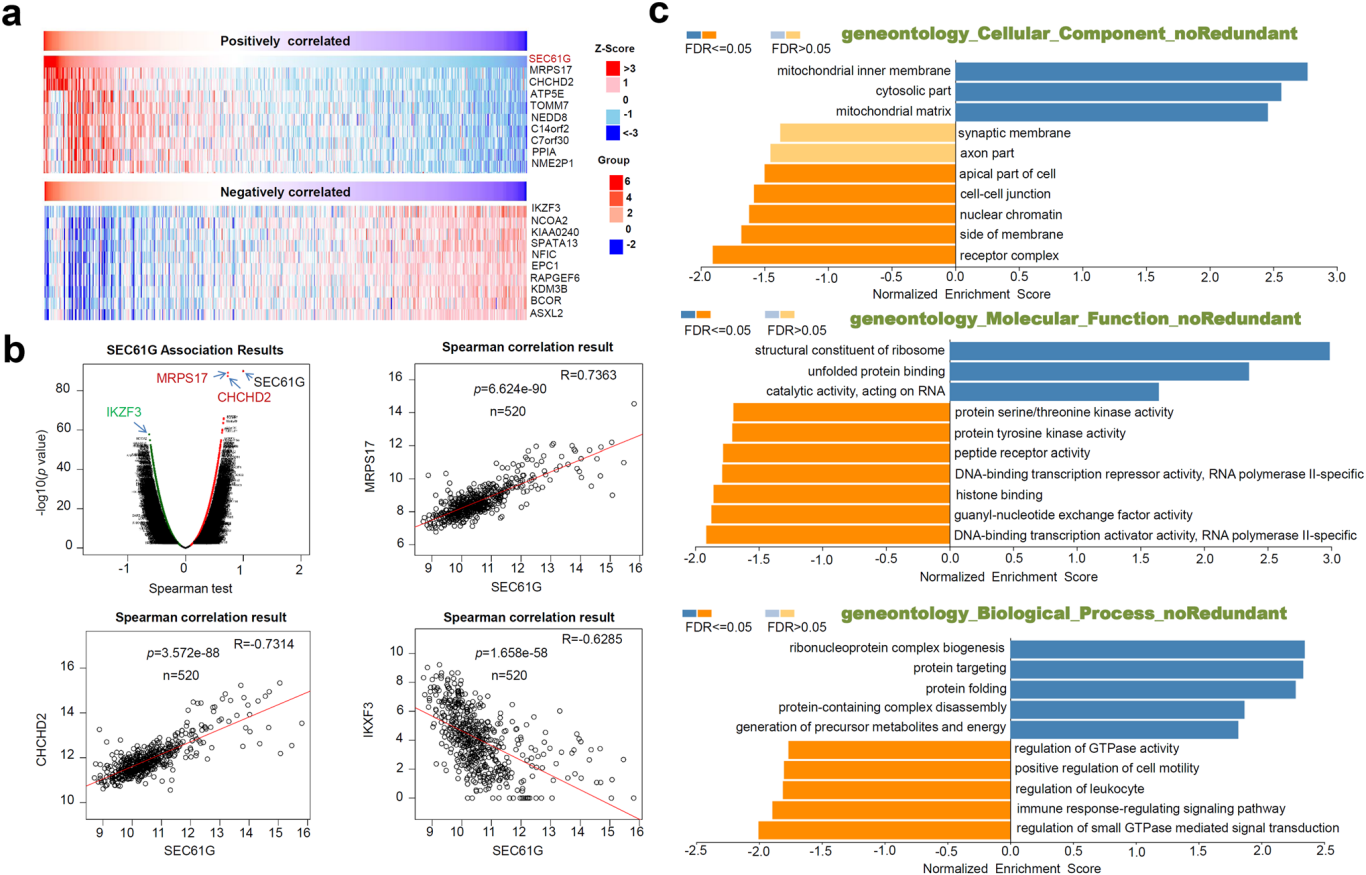
*SEC61G*-correlated gene enrichment analysis. **a** Heat map of the significant genes positively or negatively correlated with *SEC61G*. **b** Expression correlation between *SEC61G* and the selected genes in a Spearman correlation test. **c** GO enrichment analysis

To further explore the molecular mechanism of the *SEC61G* gene in tumorigenesis, we sought to identify *SEC61G*-correlated genes for pathway enrichment analysis using the LinkedOmics approach. The heat map in Fig. 4a shows the top ten significant genes positively (e.g., *MRPS17*, *CHCHD2*, etc.) or negatively (e.g., IKZF3, NCOA2, KIAA0240, etc.) correlated with *SEC61G* expression. Figure 4b further shows the association analysis results and Spearman’s correlation analysis results for *SEC61G* and *MRPS17* (*p* = 6.624e-90, *R* = 0.7363), *CHCHD2* (*p* = 3.572e-88, *R* = 0.7314), and *IKXF3* (*p* = 1.658e-58, *R* = 0.6285).

**Fig. 5:**
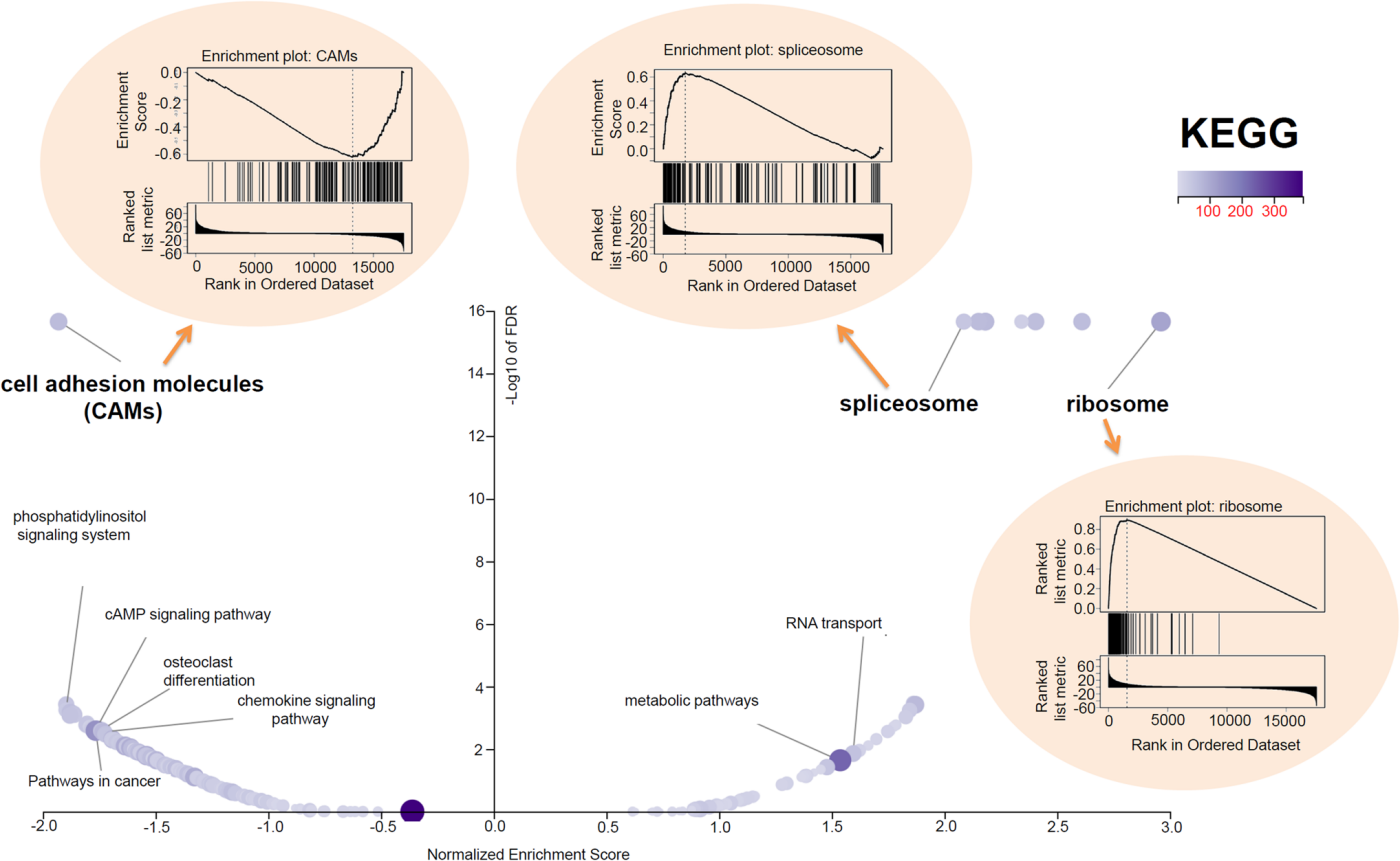
*SEC61G*-correlated KEGG pathway enrichment analysis. GSEA data of “cell adhesion molecules”, “spliceosome”, and “ribosome”

**Fig. 6:**
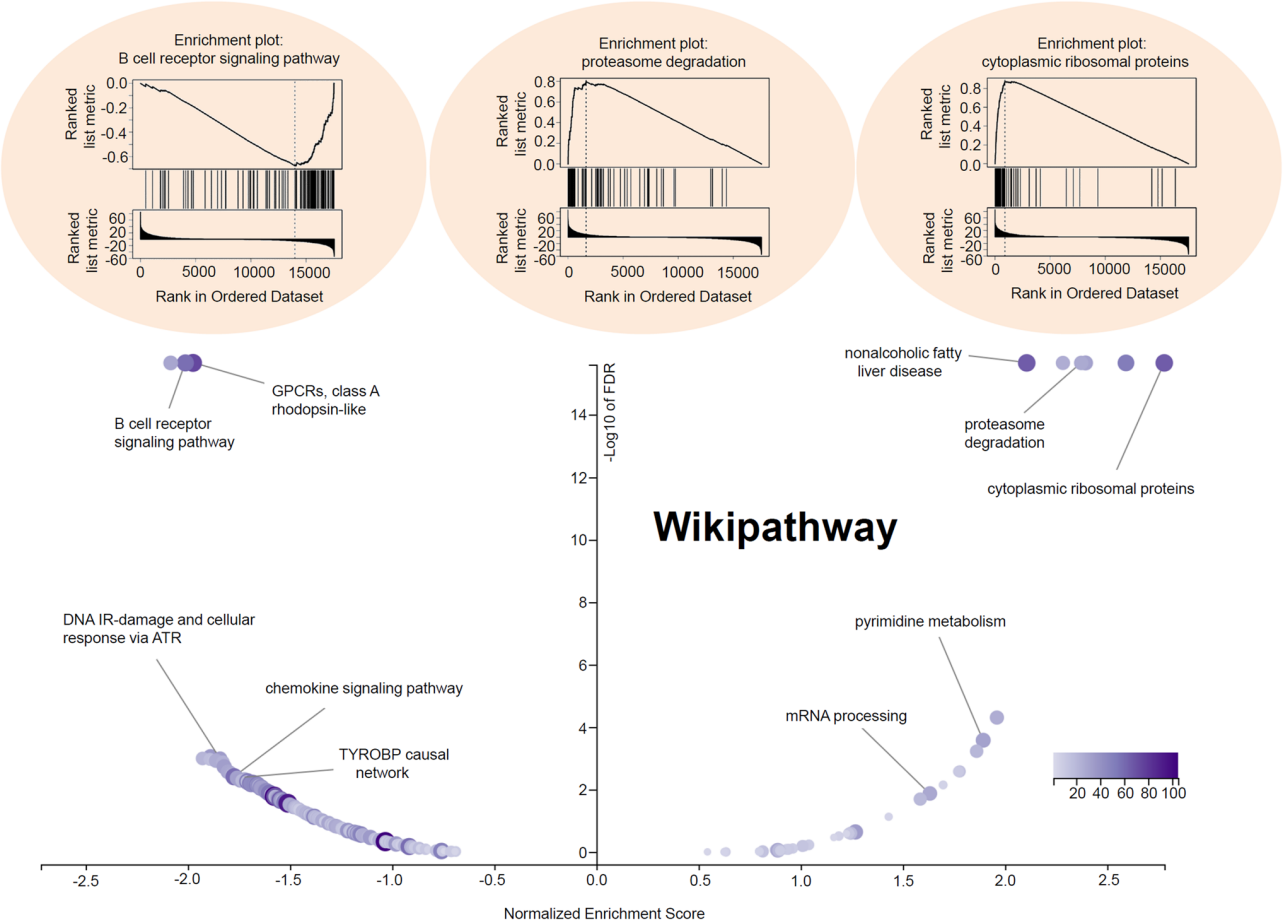
*SEC61G*-correlated Wikipathway enrichment analysis. GSEA data of the “B cell receptor signaling pathway”, “proteasome degradation”, and “cytoplasmic ribosomal proteins”

Additionally, GO enrichment, KEGG pathway, WikiPathway, and GSEA profile analyses were performed. As shown in Fig. 4c, we detected enriched cellular components (e.g., mitochondrial inner membrane or receptor complex), molecular functions (e.g., structural constituent of the ribosome or DNA-binding transcription activator activity, RNA polymerase II-specific), and biological processes (e.g., ribonucleoprotein complex biogenesis or regulation of small GTPase-mediated signal transduction). The KEGG pathway and GSEA data shown in Fig. 5 further revealed that the pathways “cell adhesion molecules (CAMs)”, “Spliceosome”, or “Ribosome”. Figure 6 of WikiPathway data also presents the enrichment plots of “B cell receptor signaling pathway”, “proteasome degradation”, and “cytoplasmic ribosomal proteins” were enriched. These results suggest that *SEC61G* participates in the pathogenesis of HNSCC, mainly through playing potential regulatory roles in the spliceosome, ribosome, proteasome degradation, immune cell regulation, and cell adhesion processes.

### Immune infiltration analysis

**Fig. 7:**
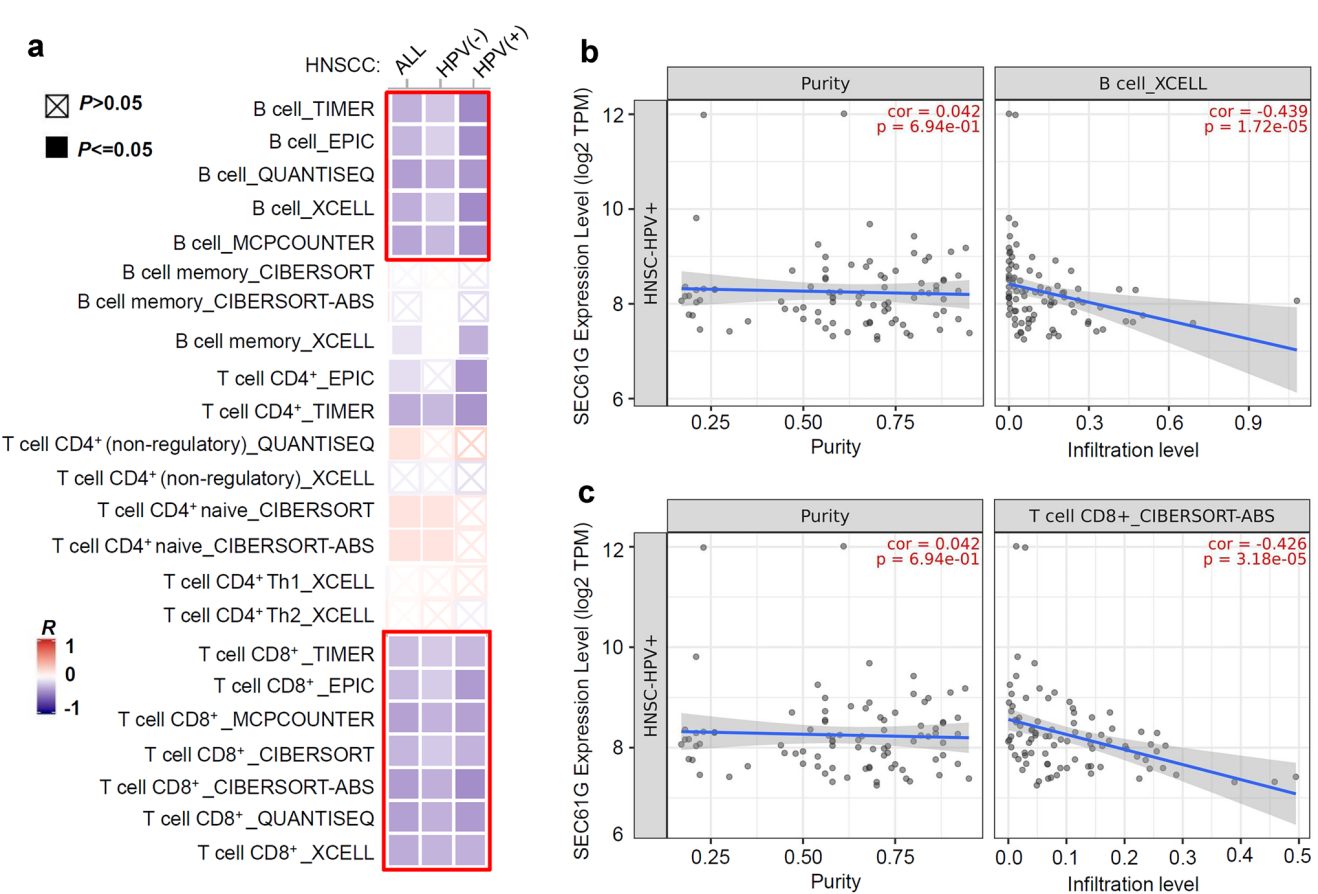
Correlation analysis between *SEC61G* expression and immune cell infiltration in HNSCC. **a** Based on the HNSCC dataset of TCGA, the potential association between *SEC61G* expression and the infiltration status of B and T immune cells was studied via TIMER2.0. **b** One scatter plot of B cells or CD8^+^ T cells is shown as an example

Finally, we utilized the TIMER, EPIC, QUANTISEQ, XCELL, MCPCOUNTER, CIBERSORT, and CIBERSORT-ABS algorithms to evaluate the potential association between *SEC61G* expression and the infiltration of different immune cells based on the HNSCC dataset in the TCGA database. Following several analyses, we observed a significant negative correlation between the immune infiltration of B cells and CD8^+^ T cells and *SEC61G* expression in all HNSCC tumors, especially HNSCC-HPV (+) tumors (Fig. 7a). The scatter plot data showing B cell infiltration with the XCELL algorithm is presented in Fig. 7b (*p* = 1.72e-05, cor = − 0.439), while the scatter plot data showing CD8^+^ T cell infiltration with the CIBERSORT-ABS algorithm is shown in Fig. 7c (*p* = 3.18e-05, cor = − 0.426). These results indicated a potential relationship between *SEC61G* expression and the infiltration of B and CD8^+^ T cells in the HNSCC tissues.

## Discussion

### High expression of *SEC61G*

It has been reported that there is a higher level of *SEC61G* in the tissues of glioblastoma multiforme than in those of lower-grade gliomas [16, 17]. In the current study, we observed an increased level of *SEC61G* in brain or central nervous system (CNS) cancer tissues across ten studies compared with its expression level in the corresponding normal tissues. In addition to in brain tumors, we also detected a high expression level of *SEC61G* in other types of tumors. For instance, we observed high expression of the *SEC61G* gene in kidney cancer tissues in six studies. Using the TCGA and Oncomine datasets, we analyzed the expression levels of *SEC61G* in normal and HNSCC tissues. In the TCGA database, compared with the 40 normal tissues samples, *SEC61G* was highly expressed in 520 primary HNSCC tissue samples, mainly including 133 tongue tumor samples and 117 larynx tumor samples. In the Oncomine database, only five datasets containing four tongue carcinomas and one oral cavity carcinoma were included. We also observed high SEC61G expression in these tumor tissues compared with that in normal tissues. Thus, these results suggest that *SEC61G* is not a tumor-specific highly expressed gene.

### CNA and *SEC61G*

*SEC61G* expression was not closely related to DNA copy number in 7p11.2 amplified oral squamous cell carcinoma (OSCC) [18]. However, genomic copy-number aberrations of *SEC61G* seem to be associated with the expression of *SEC61G* in gastric cancer (*R* = 0.93, *p* < 0.01) [19]. The data from a GWAS (genome-wide association study) indicated that single-nucleotide polymorphisms (SNPs) in *SEC61G* are associated with susceptibility to clinical SLE (systemic lupus erythematosus) [20]. In the present study, CNA of SEC61G was found to be linked to its mRNA expression level and patient overall survival rates and progression-free intervals. Furthermore, we observed a potential correlation between *SEC61G* CNA and the expression of CCT6A or HUS1. CCT6A is a member of the chaperonin-containing TCP1 complex and was reported to be associated with transcriptome reprogramming by HNSCC-derived exosomes [21]. HUS1, an important DNA damage checkpoint, participates in the regulation of the cell cycle [22, 23]. HUS1 can act as a potential tumor suppressor of hepatocellular carcinoma [24]. However, there is still no report regarding the potential correlation between HUS1 expression and oncogenesis in HNSCC. *SEC61G* CNA may be the downstream functional target of CCT6A or HUS1. This potential mechanism deserves future explorations.

### Cell biological events and *SEC61G*

As *SEC61G* is located in the membrane structure of the ER [1, 2], regulation of the biology of the ER is the most critical mechanism of cellular *SEC61G*. Nevertheless, there is limited evidence to suggest that there is a correlation between *SEC61G*-mediated ER function alterations and the occurrence of clinical tumor diseases. It was reported that the response to ER stress might contribute to the oncogenic role of *SEC61G* in glioblastoma [17]. Herein, we carried out a series of *SEC61G*-correlated gene enrichment analyses and identified a group of biological structures or processes associated with *SEC61G*, such as the mitochondrial inner membrane or receptor complex, regulation of small GTPase-mediated signal transduction, CAMs, spliceosome, and proteasome degradation, apart from ER-associated ribosome aberrations. Tumor-infiltrating immune cells, as prominent components of the tumor microenvironment, are closely linked to the initiation, progression, or metastasis of cancer [25, 26]. In the present study, we also utilized different algorithms to observe the potential association between *SEC61G* expression and the infiltration of B cells and CD8^+^ T cells in HNSCC. These findings offer a reference or possible future investigation directions for the in-depth elucidation of the molecular mechanisms underlying the oncoprotein role of SEC61G.

### HPV infection and *SEC61G*

There exists a potential correlation between *SEC61G* expression and the outcome of temozolomide treatment and radiotherapy for glioblastoma patients [8]. Due to limited clinical evidence, we could not perform a relative analysis regarding the association between *SEC61G* expression and the sensitivity of HNSCC to radiotherapy and chemotherapy. Human HPV infection is associated with the effect of clinical radiotherapy and chemotherapy in head and neck cancer patients [27–29]. Among the TCGA HNSCC samples, the HNSCC-HPV (+) group mainly consisted of 23 tonsil, 10 base of tongue, and 3 larynx tissues, while the HNSCC-HPV (−) group mainly contained 23 oral tongue and 18 larynx tissues. The expression level of *SEC61G* in the HNSCC-HPV (+) group was lower than that in the HNSCC-HPV (−) group. We also observed a relatively lower proportion of “arm-level gain” and “high amplification” copy-number variants in the HNSCC-HPV (+) group compared with the HNSCC-HPV (−) group. These results suggested a potential association between HPV infection and *SEC61G* CNA. In addition, we observed a relatively strong negative correlation between the immune infiltration of B cells and *SEC61G* expression in the HNSCC-HPV (+) group compared with the HNSCC-HPV (−) group. HPV infection might be involved in the potential association between *SEC61G* expression and the clinical therapeutic effect of treatments for HNSCC patients. CNA and the tumor microenvironment are possible mechanistic points to explore further.

Taken together, our findings first revealed the prognostic value of *SEC61G* for HNSCC patients. We observed a relationship between high expression of *SEC61G* and poor survival rates in HNSCC patients. The *SEC61G* gene may be implicated in the pathogenesis of HNSCC through the modulation of ribosomes, spliceosomes, proteasome degradation, cell adhesion, and the immune infiltration of B and CD8^+^ T cells. More in-depth molecular biological elucidations are needed.

## Supplementary Information

Below is the link to the electronic supplementary material.

Supplementary file1 Fig. S1 Phylogenetic tree of *SEC61G* in different species (TIF 2932 KB)

Supplementary file2 Fig. S2 Three factors were analyzed in the study of the correlation between *SEC61G* expression and the survival prognosis of TCGA HNSCC patients. a Race; b histological grade; c sex (TIF 2147 KB)
