# Supplementary figures and images for: SEC61G identified as a prognostic biomarker of head and neck squamous cell carcinoma
Source: Eur Arch Otorhinolaryngol. 2021 Jun 25;279(4):2039–48. doi: 10.1007/s00405-021-06955-7 (PMC8930941; doi:10.1007/s00405-021-06955-7)

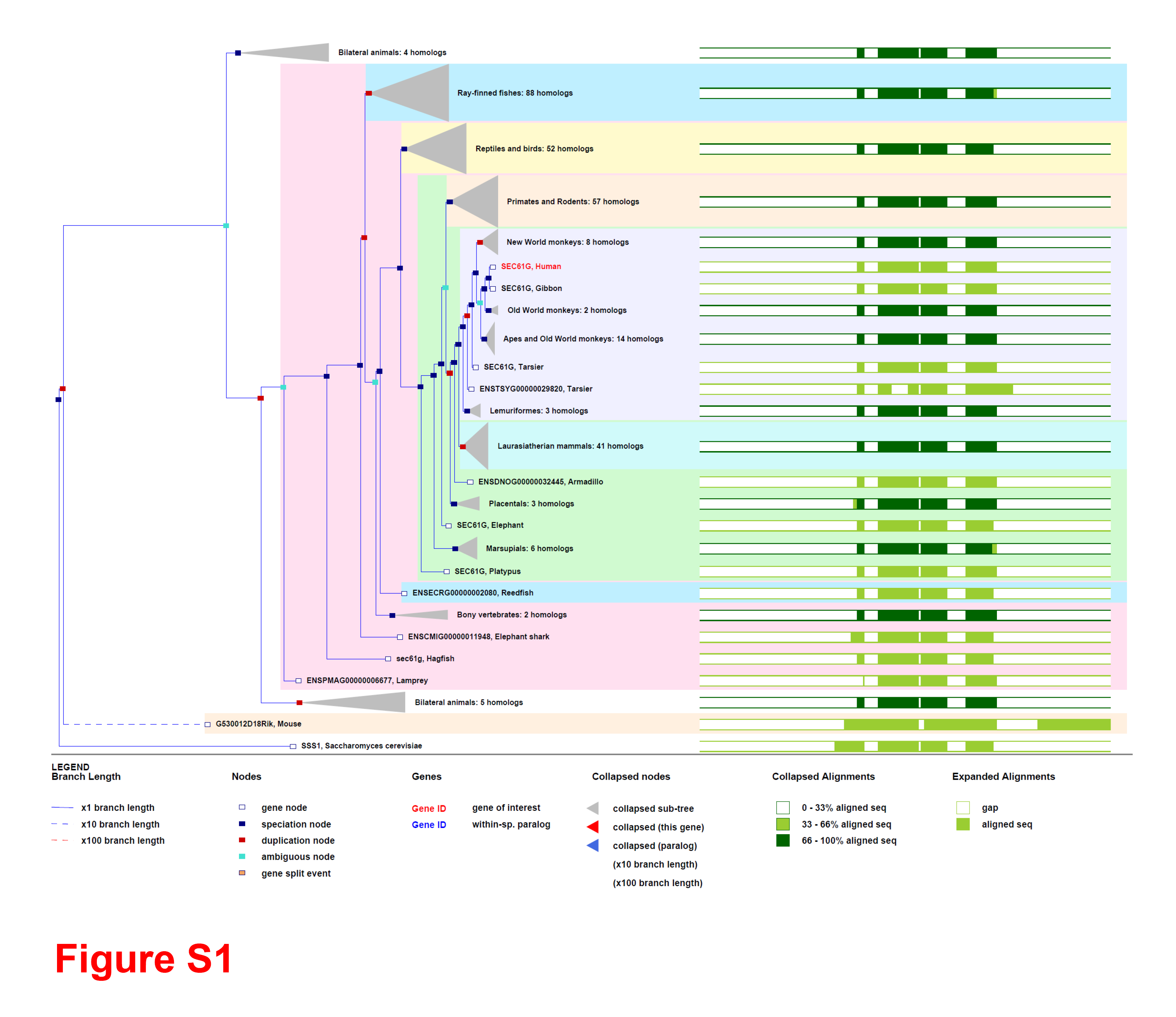

Supplement: Supplementary file 1 — Supplementary file1 Fig. S1 Phylogenetic tree of SEC61G in different species (TIF 2932 KB) [file 405_2021_6955_MOESM1_ESM.tif]

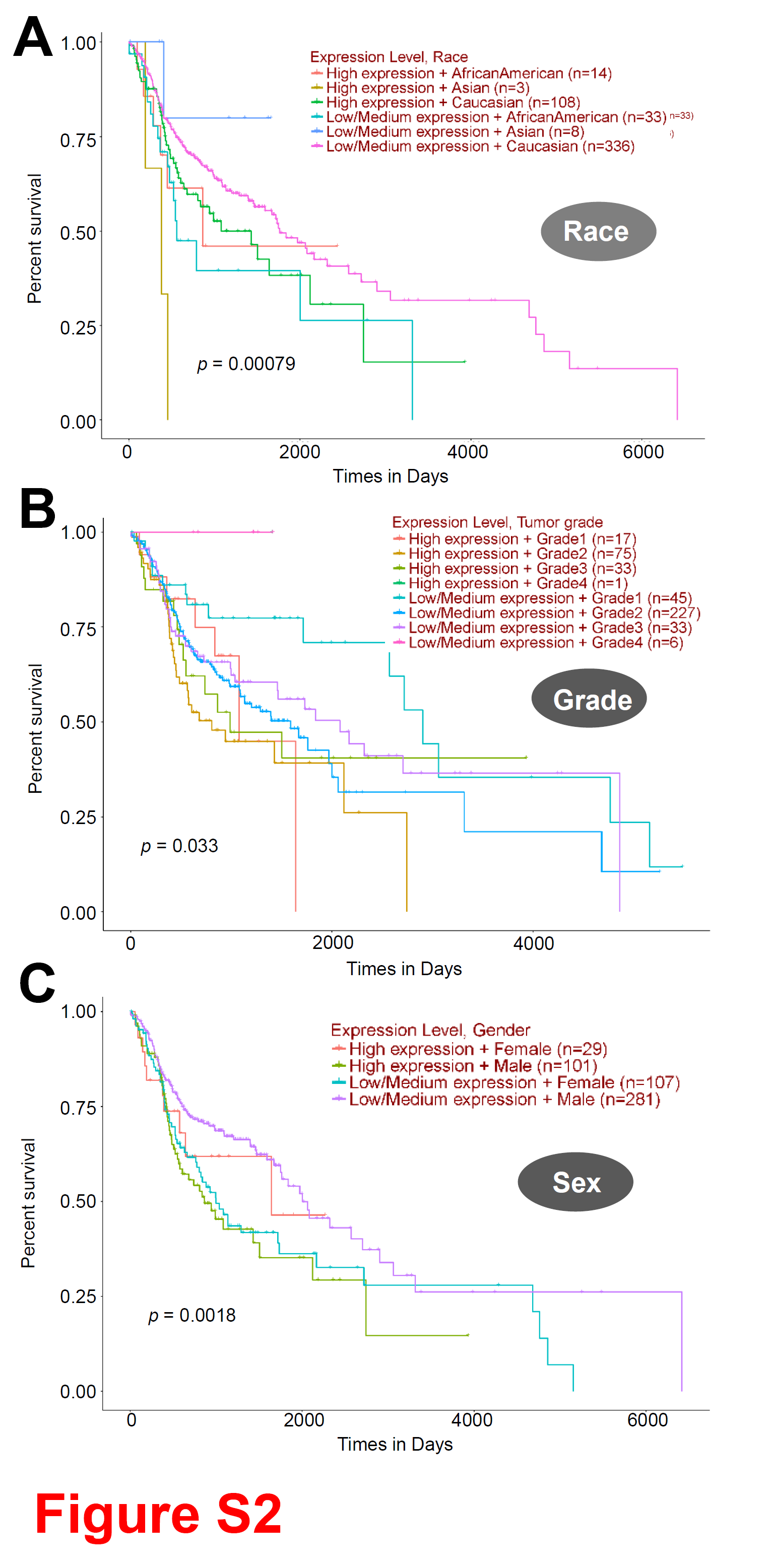

Supplement: Supplementary file 2 — Supplementary file2 Fig. S2 Three factors were analyzed in the study of the correlation between SEC61G expression and the survival prognosis of TCGA HNSCC patients. a Race; b histological grade; c sex (TIF 2147 KB) [file 405_2021_6955_MOESM2_ESM.tif]
